# Prevalence patterns and associated factors of elder abuse in an urban slum of eastern India

**DOI:** 10.1186/s12877-022-02986-9

**Published:** 2022-04-11

**Authors:** Pradnya Chandanshive, Sonu H. Subba, Swayam Pragyan Parida, Shree Mishra

**Affiliations:** 1 Department of Community Medicine and Family Medicine, All India Institute of Medical Sciences, 3rd floor, Bhubaneswar, Odisha 751019 India; 2 Department of Psychiatry, All India Institute of Medical Sciences, Bhubaneswar, Odisha India

**Keywords:** Elder abuse, Community, Urban slum, India

## Abstract

**Background:**

The prevalence of elder abuse in various parts of the world has been reported between 2.2 and 90.4%. According to some studies conducted in India, elder abuse prevalence ranges between 9.6 to 61.7%. Yet, elder abuse is an underreported issue. Most available evidence shows the involvement of close family members and caregivers in the abuse of older adults. Several factors associated with various forms of elder abuse need to be studied further. This study has attempted to capture the prevalence, pattern and associated factors of elder abuse in urban slums.

**Methods:**

This study was a cross-sectional community-based study conducted between August 2019 to August 2020 in an urban field practice area of the All India Institute of Medical Sciences, Bhubaneswar. It covers four wards of the Bhubaneswar Municipal Corporation, served by the Urban Primary Health Centre, IRC Village, Nayapalli. A total of 360 participants aged 60 years and above were included in this study. They were interviewed using various semi-structured interview schedules. Validated study tools such as Activities of Daily Living (ADL- Barthel Index), Hindi Mental Scale Examination (HMSE), Geriatric Depression Scale (GDS), and Vulnerability to Abuse Screening Scale (VASS) were also used to assess various factors.

**Results:**

Approximately one in five (19.4%) older adults reported some form of abuse. The types of elder abuse reported were physical abuse in 12 (3.3%), verbal abuse in 25 (6.9%), emotional abuse in 40 (11.1%), and financial abuse in 15 (4.2%) older adult participants. The sons and daughters-in-law of the older adult participants were the main perpetrators of abuse reported. Depression and past history of abuse were found significantly associated with any type of abuse.

**Conclusions:**

The prevalence of elder abuse in this study was considerably high, with 70 (19.4%) out of the 360 participants reporting some form of abuse. Emotional abuse was the most reported, and physical abuse was the least reported type. The most common perpetrators were those on whom the older adults depended, like their sons and daughters-in-law.

## Introduction

The rise in the proportion of the older adult population (persons aged 60 years and above) has led to population ageing [1]. The population of senior citizens comprised 8.1% of the total population of India as of 2018, which is a 2% increase over the previous 1991 census [2]. This population ageing has further caused an increased burden of health-related and social issues among the older adult. In Indian settings, the common problems reported in old age include economic issues, health-related issues, psychological issues, and elder abuse [3].

The World Health Organization defines elder abuse as ‘A single, or repeated act, or lack of appropriate action, occurring within any relationship where there is an expectation of trust which causes harm or distress to an older person’ [4]. Generally, elder abuse includes the following categories: a) Physical abuse – the infliction of pain or injury, physical coercion, or physical or drug-induced restraint; b) Psychological or emotional abuse – the infliction of mental anguish; c) Financial or material abuse – the illegal or improper exploitation or use of funds or resources of the older person; d) Sexual abuse – non-consensual sexual contact of any kind with the older person; e) Neglect – the refusal or failure to fulfil a caregiving obligation. Elder abuse may or may not involve a conscious and intentional attempt to inflict physical or emotional distress on the older person [5].

Globally, one in every six older adults faces abuse [6]. The prevalence of elder abuse in various parts of the world is anywhere between 2.2 and 90.4% [7–22]. According to a few studies done in India, it ranges from 9.6 to 61.7% [23–32]. However, the actual figures may be much higher than reported. Elder abuse is understood to be an underreported issue, as only one in every twenty-four cases are reported [6]. Various studies have revealed the close family members of the older adults as their common abusers [33–37]. Few studies on elder abuse were conducted in urban community settings, especially the slum areas. The slum regions are unfavourable for living due to various issues, such as overcrowding, nuisance of pests, poor water & sanitation facilities, inadequate ventilation and lighting [38, 39]. In addition to the dearth of studies on elder abuse in Indian slums, fewer studies have included a comprehensive assessment for association with risk factors. Hence, this study has attempted to estimate the prevalence of elder abuse in the slums. This study also aims to find the associated risk factors.

### Objectives

1. To estimate the prevalence and pattern (prevalent types) of elder abuse in urban slums of Bhubaneswar.
2. To determine the factors associated with elder abuse.

## Material and methods

### Study setting

This was a cross-sectional study conducted between August 2019 to August 2020 in the urban field practice area of the Department of Community Medicine and Family Medicine, All India Institute of Medical Sciences (AIIMS), Bhubaneswar, based in the urban slum areas of Bhubaneswar, situated in the Khordha district of Odisha. The state of Odisha is located in the eastern part of India, having a population of approximately 42 million inhabitants, comprising 30 districts [40, 41]. The Khordha district has ten blocks with a total population of 2.25 million inhabitants. The city of Bhubaneswar is the state capital, consisting of a municipal corporation with 67 administrative wards and a population close to a million (0.885 million) inhabitants [42, 43]. The study area included four wards (Ward [25, 27, 28, 37]) out of the total 67 wards of the Bhubaneswar Municipal Corporation, catered to by the Urban Primary Health Centre (UPHC), IRC Village, Nayapalli. In all, there are 19 slums with a total population of 16,863 (approximately 0.016 million) inhabitants, catered to by the UPHC, Nayapalli [42].

### Study participants

We included older adults aged 60 years and above residing for at least a year in the study area. A total of 360 study participants were randomly selected from a sampling frame prepared with over 500 participants. If a participant was unavailable at their address, the next participant on the randomly generated list was selected. Those who were not willing to participate, hearing or speech impaired, or suffering from memory loss, were excluded from the study. Hearing and speech were assessed while gathering response for sociodemographic data, with the help of a valid/official disability certificate provided to the older adult. To avoid misinterpretation of information, participants with hearing and speech impairment were excluded. None of the included participants left the study midway. The sample size for the study was calculated using the 2018 HelpAge India Survey on Elder Abuse as the reference study, which reported a prevalence of elder abuse of 25% at the country level [12].

**Fig. 1:**
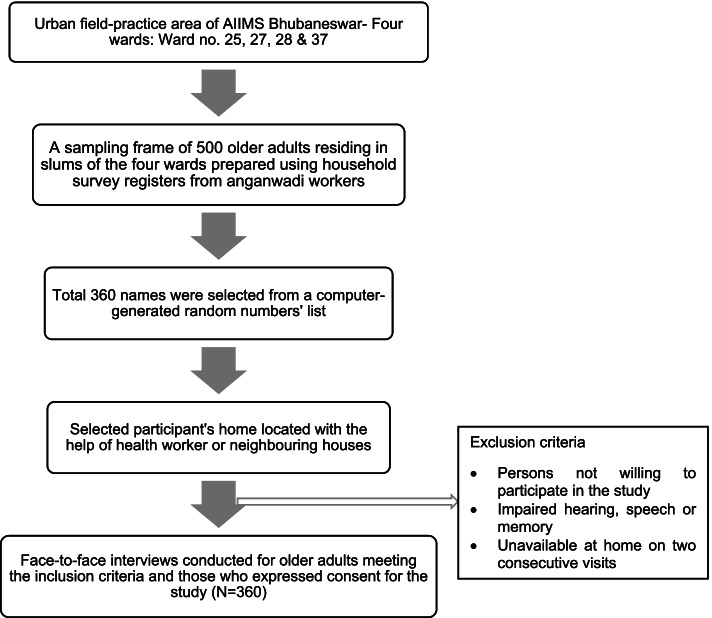
Flowchart of methodology

Using the formula, *N* = [(1.96)^2^(p*q)]/d2, where *N* = required sample size, *p* = proportion of older adults who faced abuse, q = (1-p), and d = absolute precision = 0.5, and adding a non-response rate of 20%, the sample size was (N) calculated to be 360. A sampling frame was prepared using the household survey registers from the respective slum Anganwadi centres. The study participants were chosen using simple random sampling using their serial number in the survey registers. For the required sample size, 360 older adults were selected from the sampling frame using a computer-generated list of random numbers. The house of each selected older adult was located with the help of Anganwadi workers, ASHAs or neighbouring homes in the area (Fig. 1).

### Data collection

The tools were a semi-structured interview schedule for sociodemographic variables, morbidity profile and history of abuse, the Hindi Mental Scale Examination (HMSE)- English translation, the Activities of Daily Living scale (ADL- Barthel Index), the Geriatric Depression Scale (GDS)-15 item version, and Vulnerability to Abuse Screening Scale (VASS). All methods were performed following the relevant guidelines and regulations of the Institutional Ethics Committee. On locating the study participant’s residence, a face-to-face interview was conducted with them at their house, ensuring adequate privacy and confidentiality during the interview. The sampling frame comprised over 500 participants. After excluding a participant, the next participant on the randomly generated list was selected. Approximately 30 out of all the approached older adults refused consent, and hence other participants were chosen randomly from the sampling frame to reach the sample size of 360. Most of those who refused consent did so due to various reasons such as lack of time, hesitation to provide personal information, presence of other family members, etc.

Data was entered in online EpiCollect5 forms. Those participants detected with depression were referred to the Psychiatrist consultant through free Government health services at the Urban Primary Health Centre (UPHC), Nayapalli OPD or to the Department of Psychiatry, All India Institute of Medical Sciences, Bhubaneswar. The older adult participants who reported abuse during the interview were asked if they wished to report the issue to legal authorities or other agents, and provided the Senior Citizen Helpline number. Almost all the older adults chose not to take any legal action.

There were a total of 90 questions in the interview. Each interview took 20 to 30 min. In cases with history of any abuse, an extra 10 to 15 min were taken to ensure help to that the older person. The type of questions (along with their count) were as follows: I. Socio-demographic profile (11 questions); II. Morbidity profile (10 questions); III. Activities of daily living -Barthel Index (10 questions); IV. Hindi Mental State Examination (23 questions); V. Geriatric Depression Scale (15 questions- GDS 15); VI. Vulnerability to Abuse Screening Scale (12 questions); VII. Questions specific to abuse: i. Physical abuse (2 questions), ii. Verbal abuse (2 questions), iii. Emotional/psychological abuse (3 questions), iv. Financial abuse (2 questions). A positive response to any of the abuse-specific questions was considered as a case of abuse. Few examples of the questions from the interview schedule are as follows:

Examples of interview questions:

1. *Have you been hit/beaten? (Physical abuse)*

*a. Ever 1. Yes 2. No.*

*b. In last 1 year 1. Yes 2. No.*

*2. Has anyone talked to you in an abusive language or in a rude way? (Verbal abuse).*

*a. Ever 1. Yes 2. No.*

*b. In last 1 year 1. Yes 2. No.*

*3. Are you allowed to meet your friends/family? (Emotional/psychological abuse).*

*1. Yes 2. No.*

*4. Are you allowed to interact with guests visiting your house? (Emotional/psychological abuse) 1. Yes 2. No.*

*5. Do you get enough to spend on yourself? (Emotional/psychological abuse).*

*1. Yes 2. No.*

### Data analysis

The data was collected and entered in Microsoft Excel 2010. The data obtained was analysed using Statistical Package for the Social Sciences (SPSS) software- Version 22.0. The descriptive statistics for all categorical variables were presented as percentages or proportions, and those for continuous variables as mean and standard deviation in the results. The odds ratio was calculated with a confidence interval of 95%. Binary and multivariable logistic regression analysis, Chi-square or Fisher’s exact test were also performed with a *p*-value less than 0.05 considered significant.

## Results

### Sociodemographic characteristics

Out of the total 360 study participants, 252 (70.0%) were aged 60 to 69 years. The mean age of the participants was 66.6 ± 7.8 (Mean ± SD) years. The proportion of females and males in the study was almost equal, with 193 (53.6%) female and 167 (46.4%) male participants. Only 13 (3.6%) were separated from their spouse, 133 (36.9%) widow/widower, while 214 (59.4%) of the older adults were currently married. Out of 360, 256 (71.1%) were currently unemployed, and 222 (61.7%) were illiterate.

As for the living status, 339 (94.2%) older adults were staying with their family members, while 19 (5.3%) were staying alone and one person (0.3%) each was staying with distant relatives and a rented-house roommate. The family was of extended type for 197 (54.7%), nuclear for 139 (38.6%) and joint family for 24 (6.7%) of the participants. Only 146 (40.6%) older adults availed the Odisha State Government’s social benefit scheme ‘Madhu Babu Pension Yojana’ for older persons aged 60 years and above. Smokeless tobacco was consumed by 194 (53.8%) older adults, alcohol by 18 (5.0%) and one (0.3%) admitted using illicit drugs (Table 1).

**Table 1:** Socio-demographic characteristics of older adult participants (*N* = 360)

Characteristics	Total n (%)
**Age**	
60 to 69 years	252 (70.0)
70 to 79 years	69 (19.2)
80 years and above	39 (10.8)
**Marital status**	
Married	216 (60.0)
Separated	11 (3.1)
Widow/widower	133 (36.9)
**Education status**	
Illiterate	222 (61.7)
Primary school (Grade 1 to 4)	69 (19.2)
Middle school (Grade 5 to 8)	31 (8.6)
High school (Grade 9 to 10)	28 (7.8)
Diploma/intermediate	4 (1.1)
Graduate	6 (1.6)
**Current occupation**	
Self-employed	82 (22.8)
Government	6 (1.7)
Private	16 (4.4)
Unemployed/not working	256 (71.1)
**Monthly per capita income (in Rupees) of the family**	
Below 1050	24 (6.7)
1050–2101	12 (3.3)
2102–3503	22 (6.1)
3504–7007	95 (26.4)
7008 and above	207 (57.5)
**Currently living arrangement**	
Alone	19 (5.3)
Close family members	339 (94.2)
Distant relatives	1 (0.3)
Roommate	1 (0.3)
**Type of family**	
Nuclear	139 (38.6)
Extended (Three-generation family)	197 (54.7)
Joint	24 (6.7)
**House ownership**	
Self-owned	287 (79.7)
Rented	73 (20.3)
**Availed social benefits**	
None	214 (59.4)
Madhu Babu Pension Yojana	146 (40.6)
**Substance use**^a^	
None	173 (48.0)
Alcohol	18 (5.0)
Smoking (Bidi/Cigarette)	17 (4.7)
Smokeless tobacco	194 (53.8)
Other illicit drugs	1 (0.3)

^a^Multiple responses possible

### Prevalence and pattern of elder abuse

Overall, 70 (19.4%) out of the 360 older adults faced at least one form of abuse in the past 1 year. Physical abuse was reported by 25 (6.9%) ever in the past and by 12 (3.3%) participants in the past 1 year. Nine (36.0%) out of the ever-abused older adults reported physical abuse in the past 1 year. Four (33.3%) of the 12 recently-abused older adults reported their son, and an equal number reported their daughter-in-law as the perpetrators of physical abuse. Notably, two (16.6%) of the 12 recent victims of physical abuse also reported being physically abused by their wife. One (8.3%) participant each also reported their husband, son-in-law, grandson and brother as the perpetrator of physical abuse.

Emotional/psychological abuse was reported by 40 (11.1%) of the older adult participants. Among the emotionally abused older adults, seven (17.5%) reported not having the freedom to go out of their house, 6 (15.0%) each were reportedly not free to visit friends or family or interact with guests. Ten (25%) older adult reported feeling isolated. Five (1.4%) participants reported feeling that nobody wants them around.

**Fig. 2:**
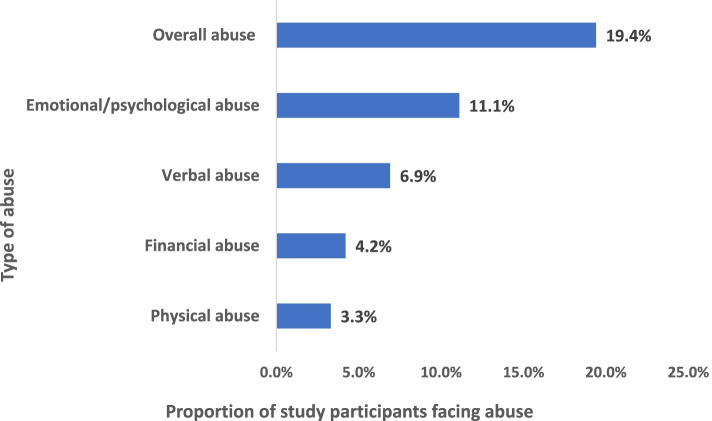
Elder abuse according to types of abuse in the study participants (*N* = 360)

Fifteen (4.2%) participants had reported some kind of financial abuse, while 345 (95.8%) did not (Fig. 2). Twenty-three participants (6.4%) out of the total 360 had faced two or more types of abuse, i.e. polyvictimization in the past 1 year.

### Mental and functional status

All the 360 (100.0%) participants had a normal HMSE score (cut off score = 24). The mean HMSE score was 29.39 ± 1.4 (Mean ± SD). Out of 360 participants, a normal score was found for 317 (88.1%) on screening for geriatric depression using the Geriatric Depression Scale by Yesavage et al. (1963). For 40 (11.1%) of the participants, the score was between 6 to 9, which is suggestive of depression and three (0.8%) participants had a GDS score ≥ 10, which is indicative of depression. None of the participants had severe (score between 21 and 60) or total (score 0 to 20) dependency. The mean ADL score was 98.8 ± 3.1 (Mean ± SD). There were 38 (10.5%) participants who were found vulnerable to abuse on screening, while the remaining 322 (89.4%) participants were not vulnerable to abuse.

### Associated factors for elder abuse

Although a majority of nineteen (27.5%) out of all older adults belonging to the 70–79 years age group had a history of at least one kind of abuse, this association was not found to be significant. No significant association was found for elder abuse with gender, marital status, education status, employment status, household type, current living arrangement, house ownership, per capita monthly income, availing social schemes, chronic illness history, substance use, ADL score and VASS score (Table 2).

**Table 2:** Association of factors with overall abuse (at least one type of abuse) (*N* = 360)

Characteristics	Any type of abuse			
	Yes	No	Total	***p***-value
**Age (years)**				
60–69	43 (17.1)	209 (82.9)	252	χ^2^ = 3.82
70–79	19 (27.5)	50 (72.5)	69	*p* = 0.15
80 & above	8 (20.5)	31 (79.5)	39	
**Gender**				
Male	36 (21.6)	131 (78.4)	167	χ^2^ = 0.89
Female	34 (17.6)	159 (82.4)	193	*p* = 0.35
**Marital status**				
Widow/widower	20 (15.0)	113 (85.0)	133	Fisher’s exact = 2.73
Separated	2 (18.2)	9 (81.8)	11	*p* = 0.25
Married	48 (22.2)	168 (77.8)	216	
**Education status**				
Illiterate	38 (17.1)	184 (82.9)	222	
Primary school	16 (23.2)	53 (76.8)	69	Fisher’s exact = 4.63
Middle school	7 (22.6)	24 (77.4)	31	*p* = 0.42
High school	8 (28.6)	20 (71.4)	28	
Diploma/intermediate	1 (25.0)	3 (75.0)	4	
Graduate	0 (0.0)	6 (100.0)	6	
**Occupation**				
Unemployed	55 (21.5)	201 (78.5)	256	Fisher’s exact = 6.55
Self-employed	13 (15.9)	69 (84.1)	82	*p* = 0.07
Government job	2 (33.3)	4 (66.7)	6	
Private job	0 (0.0)	16 (100.0)	16	
**Household type**				
Nuclear	25 (18.0)	114 (82.0)	139	Fisher’s exact = 0.38
Extended	40 (20.3)	157 (79.7)	197	*p* = 0.85
Joint	5 (20.8)	19 (79.2)	24	
**Currently living with**				
Alone	5 (26.3)	14 (73.7)	19	χ^2^ = 0.60
Family/others	65 (19.1)	276 (80.9)	339	*p* = 0.44
**Social schemes**^a^				
No	47 (22.4)	163 (77.6)	210	χ^2^ = 2.39
Yes	23 (15.8)	123 (84.2)	146	*p* = 0.12
**Chronic illness**				
No	53 (19.2)	223 (80.8)	276	χ^2^ = 0.04
Yes	17 (20.2)	67 (79.8)	84	*p* = 0.83
**Substance use**				
No	39 (22.5)	134 (77.5)	173	χ^2^ = 2.04
Yes	31 (16.6)	156 (83.4)	187	*p* = 0.15
**ADL score**				
Dependent	1 (9.1)	10 (90.9)	11	Fisher’s exact = 1.16
Slightly dependent	9 (15.5)	49 (84.5)	58	*p* = 0.56
Independent	60 (20.6)	231 (79.4)	291	
**GDS score**				
Normal (Score < 5)	56 (17.7)	261 (82.3)	317	Fisher’s exact = 5.36
Abnormal (score ≥ 5)	14 (32.6)	29 (67.4)	43	***p*** **= 0.02**
**VASS score**				
Not vulnerable	60 (18.6)	262 (81.4)	322	χ^2^ = 1.28
Vulnerable to abuse	10 (26.3)	28 (73.7)	38	*p* = 0.28

^a^Social schemes: These are public welfare schemes announced by the Government of India at the Central or State levels for various cross-sections of the society. In this study, such social welfare schemes for the older population were considered, eg. Old-age pension scheme named ‘Madhu Babu Pension Yojana’ of the Odisha State Government for all persons aged ≥60 years

However, a significant proportion of older adults with a history of any form of abuse also showed signs of depression on screening with the Geriatric Depression Scale (GDS). Out of those participants who had a normal GDS score, 56 (17.7%) had faced elder abuse. In contrast, out of those older adults having an abnormal score (5 and above) suggestive or indicative of depression, 14 (32.6%) had reported facing some kind of abuse. Hence, those found with depression on screening with GDS-15 were found to be significantly more likely to experience elder abuse as compared to those found to have no depression (*p* < 0.05). On further analysis using binary logistic regression, a significant association was found between elder abuse and depression (*p* < 0.05). Those with depression were found to be over two times more likely to face elder abuse as compared to those without depression (OR = 2.30, 95% CI = 1.03–5.15) (Table 3). There was no other significant association found on binary and multivariable logistic regression analysis; hence only the binary logistic regression analysis has been presented in these results.

**Table 3:** Binary logistic regression for factors associated with elder abuse in the past year (*N* = 360)

Characteristics	OR (95% CI)	***p***-value
**Age (years)**		
60–69	Reference	–
70–79	1.61 (0.63–4.13)	0.33
80 & above	0.68 (0.25–1.84)	0.44
**Gender**		
Male	Reference	
Female	0.93 (0.52–1.66)	0.80
**Marital status**		
Widow/widower	Reference	–
Separated	1.91 (1.00–3.64)	0.05
Married	1.28 (0.25–6.68)	0.77
**Education status**		
Illiterate	Reference	–
Primary school	1.87 (0.20–17.52)	0.58
Middle school	2.56 (0.27–24.00)	0.41
High school	2.01 (0.19–20.96)	0.56
Diploma and above	2.69 (0.26–27.81)	0.41
**Occupation**		
Unemployed	Reference	–
Self-employed	3.40 (0.69–16.81)	0.13
Government/Private job	2.52 (0.46–13.70)	0.28
**Household type**		
Nuclear	Reference	–
Extended	1.34 (0.41–4.34)	0.63
Joint	0.92 (0.29–2.85)	0.88
**Currently living with**		
Alone	Reference	–
Family/others	0.52 (0.15–1.82)	0.31
**Social schemes**		
No	Reference	–
Yes	0.56 (0.29–1.05)	0.07
**Chronic illness**		
No	Reference	–
Yes	0.98 (0.51–1.90)	0.95
**Substance use**		
No	Reference	–
Yes	1.55 (0.88–2.71)	0.13
**GDS score**		
Normal (Score < 5)	Reference	–
Abnormal (score ≥ 5)	**2.30 (1.03–5.15)**	**0.04**
**VASS score**		
Not vulnerable	Reference	–
Vulnerable to abuse	1.18 (0.47–2.96)	0.71

## Discussion

### Prevalence and pattern of elder abuse

The present study estimated the prevalence and determinants of elder abuse in urban slums of Bhubaneswar city and found that 70 (19.4%) of its study participants reported some form of abuse (overall abuse). Among the types of elder abuse, in the past 1 year, physical abuse was reported in 12 (3.3%), verbal abuse in 25 (6.9%), emotional abuse in 40 (11.1%), financial abuse in 15 (4.2%) of the study participants. Hence, emotional abuse was the most commonly reported form of elder abuse. Out of the total 70 participants who faced at least some form of abuse, as much as 23 (32.8%) faced multiple types of abuse.

The study setting was a slum region in the urban field practice area of AIIMS, Bhubaneswar. In addition to service by the state government, i.e. Government of Odisha, the study area is also served by the Department of Community Medicine and Family Medicine of AIIMS Bhubaneswar. The slum inhabitants are generally more predisposed to health issues due to lack of sanitation and presence of overcrowding, in addition to unemployment, substance use and illiteracy, leading to overall substandard living conditions. As the setting for this study was an urban area, 34% of the older adult participants were staying in a nuclear family or only with their spouse. Owing to the stressful living conditions of the slums, the older adults may be more vulnerable to elder abuse by their family members.

The present study employed a comprehensive assessment of mental and cognitive functions to study the associated risk factors. However, due to close proximity of the houses in the slums, there is a possibility of an underreporting of elder abuse, in spite of the best possible measures undertaken for maintaining privacy and confidentiality. The investigator being a medical professional, the utmost measures were taken to provide the older adults with free medical advice and aid, ensuring cooperation and honesty considering the sensitive nature of the study.

Emotional abuse was the most reported form of abuse in this study, followed by verbal abuse. Although verbal abuse can be included in emotional abuse, in this study, it is reported separately. The various aspects of emotional abuse included in the interview were: *making the older adult feel isolated, making the older adult stay in bed even though they are not ill, preventing the older adult from visiting family/friends, making them feel worthless*, etc. The older adults might be treated as a burden by their family, especially considering the stressful substandard lifestyle of the slum areas, which might contribute to emotional abuse [7, 26, 44].

Verbal abuse was considered as use of insults, humiliating terms, hurtful language, etc. The dependent nature of most of the participants in this study due to lack of a source of income, chronic illnesses, illiteracy, etc. might have been contributory to them facing verbal abuse. The same also possibly played a role in physical abuse. Financial abuse, however, might have been the least reported due to poor socioeconomic status of most of the older adult participants.

Similar to the present study, Yon et al. (2017) reported a pooled overall abuse prevalence of 15.7% across 28 countries in their systematic review [19]. Their study had included community-based studies reporting the past-year prevalence of abuse in older adults aged 60 years and above. Their study had also been conducted in the community. Hence, the similar overall prevalence of abuse might be because their study inclusion criteria were the same as the present study’s. On the other hand, the systematic review findings by Dong et al. (2015) revealed a range of 2.2 to 61.1% for elder abuse across countries in Europe, America and Asia [8]. However, their review included studies conducted in the community as well as hospital settings, across various countries, hence the variation in the prevalence of abuse.

In contrast, two separate systematic reviews on studies from Iran found a prevalence of abuse much higher than that of the present study. The reported pooled prevalence of overall abuse was 48.3 and 45.7% by Abdi et al. (2019) and Arab-Zozani (2018), respectively [14, 21]. These systematic reviews include studies conducted in Iran across urban and rural settings, in the community as well as hospitals. Hence, the prevalence of elder abuse found in the present study differs greatly from the systematic reviews of Iranian studies on elder abuse.

A prevalence of 4.5%, which is lower than the present study, was reported by the community-based Malaysian MAESTRO study by Sooryanarayana et al. (2017) [15]. Although theirs was also a community-based study in participants with the same age-cut off as the present study, their study instrument used was different. The sample size in their study was much larger than that in the present study. Another study from Asia by Chokkanathan et al. (2018) also reported a lower prevalence of 8.3% for overall abuse in a community in Singapore [11]. However, their study used convenience sampling in a residential area with a high concentration of older adults. Hence, having a majority of the geriatric population in the community might have been more protective against elder abuse than the urban slum setting in the present study.

In India, the study by HelpAge India (2018) reported an overall abuse prevalence of 25% across 23 cities of India, which was slightly higher than the current study findings [24]. The prevalence of overall abuse found in Bhubaneswar city by HelpAge India study was 23%. Their study was mixed-method in nature. However, the unstructured part of the interview schedule of the present study was similar to their study questionnaire. This might explain why their findings were similar to the present study findings. Few other studies have found overall abuse prevalence similar to the HelpAge India study [26, 27, 31]. However, Kumar et al. (2019) reported a much lower prevalence than the current study with any type of elder abuse in 9.6% of their participants in an urban community from North India [29]. There was a difference in the sample size and the screening tool used in their study. While their study used only the The Hwalek-Sengstock Elder Abuse Screening Test (HS-EAST) screening tool, the present study used the VASS which is a modified version of the HS-EAST scale, in addition to specific questions for elder abuse. Another urban community-based study by Mawar et al. (2018) reported a slightly higher proportion (24.3%) than the current study [26]. Although, they used only the Vulnerability to Abuse Screening Scale (VASS) to measure elder abuse. Various factors differ from region to region and across the administrative States in India, which might contribute to the different prevalence rates in elder abuse.

A hospital-based study by Nisha et al. (2016) in Bangalore reported findings similar to the present study. They found an overall abuse prevalence of 16%, which is slightly lower than the present study [23]. Their study was conducted in a hospital-based setting in a geriatric clinic. Their participants included only those older persons who visited the hospital for medical consultation, hence missing those who might be facing neglect or emotional abuse. The study found that the number of participants reporting abuse was higher than the number of participants found vulnerable with the help of the VASS questionnaire. The VASS questionnaire consists of four domains: Vulnerability, Dependence, Dejection and Coercion. A score of 3 or more suggests vulnerability of elder abuse. However, this study has considered any positive response to the specific elder abuse questions as the cut off for elder abuse in the participants. This might be the possible reason for the discrepancy in the positive VASS score and the prevalence of elder abuse found in this study.

### Factors associated with elder abuse

There was no significant association found for overall elder abuse with any sociodemographic factors, history of chronic disease, ADL score, or the Vulnerability to Abuse Screening Scale (VASS) score. However, those with suggestive or indicative depression on screening were also significantly more than two times likely to face abuse (OR = 2.30, 95% CI = 1.03–5.15). Depression has been found as a significant predictor of elder abuse in both males and females by Dong et al. (2010) [45]. Wu et al. (2012) found depression to be a significant risk factor for physical abuse, psychological abuse and neglect [46]. Gao et al. (2018) also reported that persons suffering from depression were more likely to face elder abuse by family members [13]. On the other hand, Nisha et al. (2016) reported a significantly higher prevalence of elder abuse with the presence of moderate depression than with severe depression, which is in line with our study findings [23]. Whereas Patel et al. (2018) conducted a study with their participants being older adult patients suffering from depression and found a statistically significant association with elder abuse [27]. In community settings, Sembiah et al. (2020) and Mawar et al. (2018) also reported a significantly higher prevalence of abuse in depressed older adult [26, 31].Various other studies have found an association of elder abuse with past history of abuse [38, 47–51].

### Strengths and limitations

In the current study, we have used simple random sampling, which helps to eliminate selection bias and possible design effect. For the risk factors associated with elder abuse, morbidity profile was assessed using a self-designed questionnaire. In contrast, validated tools were applied for assessing the cognitive status (using HMSE), dependency on daily activities (Barthel index for Activities of Daily Living) as well as the 15-item Geriatric Depression Scale. Besides this, the Vulnerability to Abuse Screening Scale (VASS) was used to screen for elder abuse.

The present study had excluded older adult suffering from severe mental, visual or hearing impairment considering the exhaustive nature of the interview schedule. As there is evidence of an association between elder abuse and those physically and mentally impaired, this study might have missed a vulnerable population. Hence, the prevalence of abuse found in this study might be underestimated. Also, the family members were not interviewed, which might have provided information about caregiver-reported elder abuse. Owing to the cross-sectional nature of this study, a temporal association cannot be established between the significantly associated factors and elder abuse. There is a possibility of social desirability bias as disrespect or abuse of elders is often looked down upon in Indian society. This study managed to capture physical, verbal, emotional/psychological and financial abuse; however, neglect and sexual abuse could not be captured.

## Conclusion

The prevalence of any form of elder abuse found in this study was 19.4%, which corroborates with various Indian and global studies. The most common type of abuse was emotional/psychological abuse (11.1%), and the least was physical abuse (3.3%). The perpetrators of abuse were mostly the sons and their spouses. Depression has been found to be significantly associated with elder abuse in the present study. A previous history of physical and verbal abuse was significantly associated with recent abuse.

There is a scope for more research on elder abuse in the country, especially qualitative research, to study the factors associated with elder abuse in depth. The older adults can be empowered with vocational programmes, awareness generation regarding their issues and remedial measures available. Increasing the accessibility of existing protective mechanisms for the older adults, such as social benefits and legal provisions is need of the hour. There is a need for integration between international and national agencies to take legal and programmatic action against elder abuse. The state and district level centres can function under the national framework to include the issue of abuse of the older adults, primarily through national programmes like the National Programme for Health Care for the Elderly (NPHCE). The primary health care providers and family physicians can also play a role in identifying and resolving cases of elder abuse with adequate training and reporting mechanisms in place.

## Data Availability

The datasets used and/or analysed during the current study are available from the corresponding author upon reasonable request.

## Abbreviations

ADL: Activities of Daily Living
AIIMS: All India Institute of Medical Sciences
ASHA: Accredited Social Health Activist
AWC: Anganwadi Centre
AWW: Anganwadi Worker
BBSR: Bhubaneswar
EASI: Elder Abuse Suspicion Index
EAST: Elder Abuse Screening Test
GDS: Geriatric Depression Scale
HMSE: Hindi Mental State Examination
HS-EAST: Hwalek-Sengstock Elder Abuse Screening Test
IEC: Institute Ethics Committee
MMSE: Mini-Mental State Examination
NGO: Non-Government Organization
NPHCE: National Programme for Health Care of the Elderly
SD: Standard Deviation
SPSS: Statistical Package for The Social Sciences
UN: United Nations
UPHC: Urban Primary Health Centre
VASS: Vulnerability to Abuse Screening Scale
WHO: World Health Organization
